# Procedure for Organizing a Post-FDA-approval Evaluation of Antidepressants

**DOI:** 10.7759/cureus.29884

**Published:** 2022-10-03

**Authors:** Farrokh Alemi, Hua Min, Melanie Yousefi, Laura K Becker, Christopher A Hane, Vijay S Nori, William H Crown

**Affiliations:** 1 Health Adminstration and Policy, George Mason University, Fairfax, USA; 2 Health Administration and Policy, George Mason University, Fairfax, USA; 3 School of Nursing, George Mason University, Fairfax, USA; 4 Analytics, OptumLabs, Eden Prairie, USA; 5 Data Science, OptumLabs, Eden Prairie, USA; 6 Engineering, OptumLabs, Eden Prairie, USA; 7 Research, The Heller School for Social Policy and Management, Brandeis University, Waltham, USA

**Keywords:** cohorts, effectiveness, administrative data, antidepressants, depression

## Abstract

Purpose: The study reports the construction of a cohort used to study the effectiveness of antidepressants.

Methods: The cohort includes experiences of 3,678,082 patients with depression in the United States on antidepressants between January 1, 2001, and December 31, 2018. A total of 10,221,145 antidepressant treatment episodes were analyzed. Patients who had no utilization of health services for at least two years, or who had died, were excluded from the analysis. Follow-up was passive, automatic, and collated from fragmented clinical services of diverse providers.

Results: The average follow-up was 2.93 years, resulting in 15,096,055 person-years of data. The mean age of the cohort was 46.54 years (standard deviation of 17.48) at first prescription of antidepressant, which was also the enrollment event (16.92% were over 65 years), and most were female (69.36%). In 10,221,145 episodes, within the first 100 days of start of the episode, 4,729,372 (46.3%) continued their treatment, 1,306,338 (12.8%) switched to another medication, 3,586,156 (35.1%) discontinued their medication, and 599,279 (5.9%) augmented their treatment.

Conclusions: We present a procedure for constructing a cohort using claims data. A surrogate measure for self-reported symptom remission based on the patterns of use of antidepressants has been proposed to address the absence of outcomes in claims. Future studies can use the procedures described here to organize studies of the comparative effectiveness of antidepressants.

## Introduction

Poor treatment of depression worsens medical outcomes [1], increases the risk of suicide [2,3], increases disability [4], hastens cognitive decline/dementia [5], increases falls/injuries [6], and causes drug-drug interactions [7], and wastes health care resources [8]. Depression can affect compliance with medication; patients may abandon effective medical treatment [9]. As a consequence, it is not surprising that late-life depression has the highest mortality among all chronic comorbidities [10].

Despite, literally, thousands of randomized clinical trials [11], a great deal about the effectiveness of antidepressants is still not known, primarily because the size and composition of randomized trials limit subgroup analysis. Recent reviews of the effectiveness of antidepressants show that average differences among antidepressants across the entire population are negligible [12-15]. These studies have called for post-market release evaluation of antidepressants in large enough samples that would allow comparison of antidepressants in a variety of subgroups. This study was undertaken to address these calls to action.

This cohort was organized to understand the comparative effectiveness of antidepressants. It can help regulatory agencies, the scientific community, clinicians, and patients examine which antidepressant is best and for whom. In particular, treatment-resistant patients can search the findings from this study to identify the antidepressant most likely to address their needs.

## Materials and methods

This study used administrative claims data from the OptumLabs® Data Warehouse [16] (Optum, Inc., Eden Prairie, Minnesota, United States) to select patients (a) with major depression and related illnesses and (b) on antidepressants. It is a commercially and publicly available dataset. Informed consent was waived by the George Mason University Institutional Review Board. All methods were performed in accordance with the relevant guidelines and regulations.

Major depression was defined using International Classification of Diseases versions 10 and 9 (See Appendix for list of codes used). Antidepressants were identified using the HEDIS® National Drug Code (NDC) [17]. We used the 2019 list of codes to identify the generic names of antidepressants and then reused these names to generate the codes for earlier years. 

**Figure 1:**
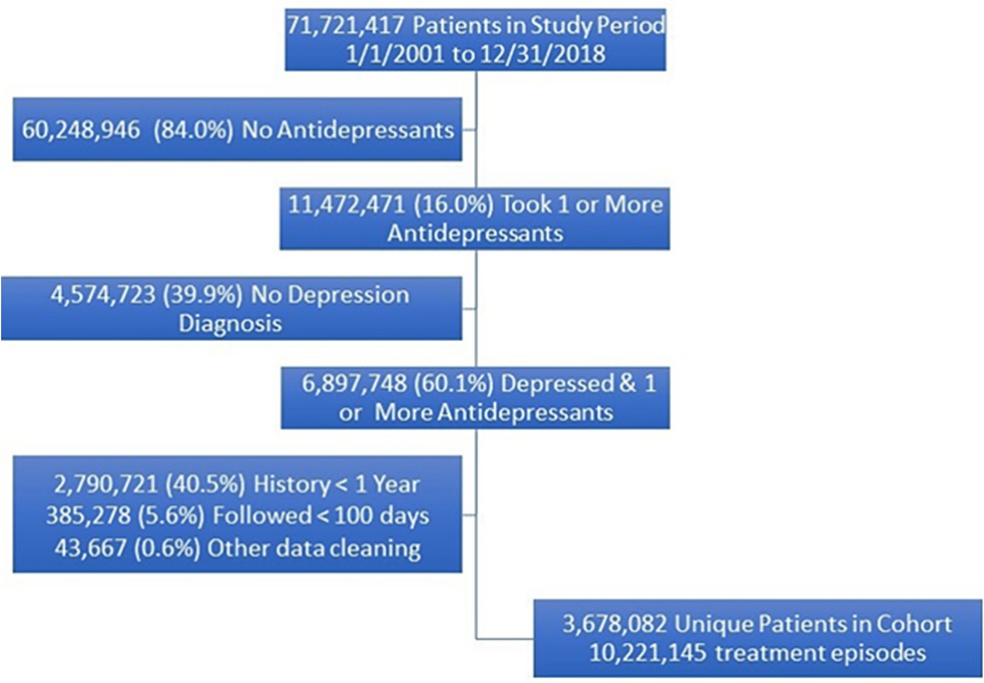
Construction of the Cohort

The study focused on enrollees eligible for insurance between January 1, 2001, and December 31, 2018 (Figure 1). We excluded 4,574,723 members who did not have a diagnosis of depression but had taken antidepressants. We excluded 2,790,721 members who were not enrolled in a health plan for at least one year prior to their first antidepressant. Additionally, 385,278 patients were excluded for having enrollment for less than 100 days after their episode started. Lastly, 43,677 (<1%) patients had anomalous birth years or other data cleaning that led to their exclusion. This resulted in 3,678,082 members being included in the cohort. They reported 10,221,145 antidepressant treatment episodes.

**Figure 2:**
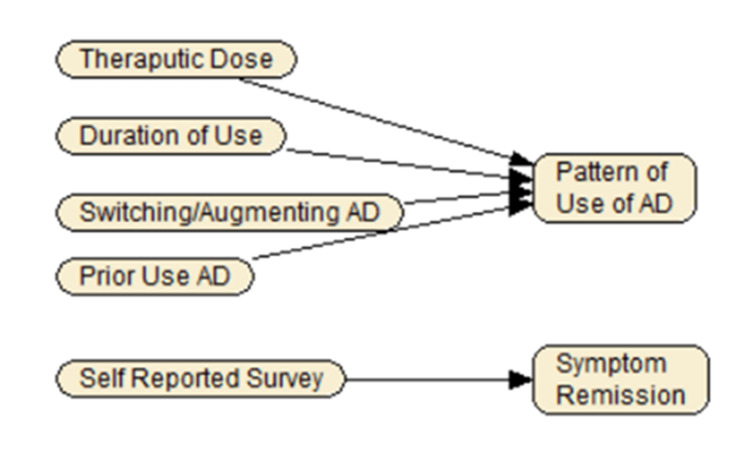
A Surrogate Measure for Self-Reported Symptom Remission Based on Patterns of Use of Antidepressants AD: antidepressants

Unfortunately, patient-reported remission of depression symptoms was not consistently available in our data; we had to find a surrogate measure based on patterns of use of antidepressants (Figure 2). We relied on: (a) duration of use, (b) reaching the therapeutic dose, (c) switching from the antidepressant to another or augmenting the antidepressant with another medication (augmentation refers to the use of another added antidepressant), and (d) use of antidepressants prior to starting this medication. During the first 100 days, it is logical that these indicators are associated with the probability of symptom remission. At the same time, there are a number of scenarios under which the use of these indicators may not be reasonable. In particular, while switches in medications can be used to judge that the initial medication was not successful, the reverse is not always true. Many patients may continue to take their medications despite a lack of adequate response. For example, nearly one-third of manic depressive patients do not achieve symptom remission but continue with their medications [18]. We checked the accuracy of our proposed surrogate measure for remission using the sequenced treatment alternatives to relieve depression (STAR*D) data available through the National Institute of Mental Health (NIMH), which included data on both patient-reported remission and antidepressant use. The findings were reassuring; the surrogate measure was a nearly perfect (area under the receiver operating curve of 0.93) predictor of patient-reported symptom remission [19]. Therefore, these five measures and the associated probability model were used as the surrogate measure for symptom remission. In addition, the patient was assumed to be in remission if clinicians had diagnosed the patient with any of the following codes, which include a reference to remission: F32.4, F32.5, F33.40, F33.41, F33.42 & 296.25, 296.35, 296.26, 296.30, and 296.36.

## Results

This cohort included 3,678,082 patients from all states in the US. The mean age of the cohort was 46.54 years (standard deviation of 17.48); 16.92% were over 65, and 6.85% were teenagers (Table 1). Due to privacy regulations, the race was assigned based on the proportion of race in the individual’s county of residence. County-based race information was available for 99.83% of individuals. The majority of the patients were predominantly White (77.24%), 14.01% Black, and 13.56% Hispanic.

**Table 1:** Demographic Distribution of Patients (Race was Inferred From County Where the Patient Resided) IQR: interquartile range

As of First Episode:	Unique Patients (N = 3,678,082)
Age	mean: 46.54; std: 17.48 median: 46.0
Age Category	
13-19	252,086 (6.85%)
20-40	1,157,601 (31.47%)
41-64	1,654,834 (44.75%)
65-79	499,249 (13.57%)
80+	123,312 (3.35%)
Gender	
Female	2,551,031 (69.36%)
Male	1,127,051 (30.64%)
Insurance	
Commercial	3,003,628 (81.66%)
Medicare Advantage	673,045 (18.30%)
Missing	1,409 (0.04%)
Race Based on County of Residence	3,672,008 (99.83%)
White	mean: 77.24; std: 14.33
Black	mean: 14.01; std: 12.29
Asian	mean: 4.40; std: 4.84
Hispanic	mean: 13.56; std: 13.87
Hawaiian	mean: 0.12; std: 0.96
Native American	mean: 1.44; std: 2.04
Other	mean: 5.50; std: 4.96
Follow-up years	mean: 2.93; std: 2.72; median: 1.98 IQR: 0.95-3.97

In order to demonstrate how a post-FDA-approval study can help in the understanding of the effectiveness of antidepressants, we present some preliminary findings from our data. In 10,221,145 episodes, within the first 100 days of start of the episode, 4,729,372 (46.3%) continued their treatment, 1,306,338 (12.8%) switched to another medication, 3,586,156 (35.1%) discontinued their medication, and 599,279 (5.9%) augmented their treatment.

Finding 1: changes in antidepressant use

**Figure 3:**
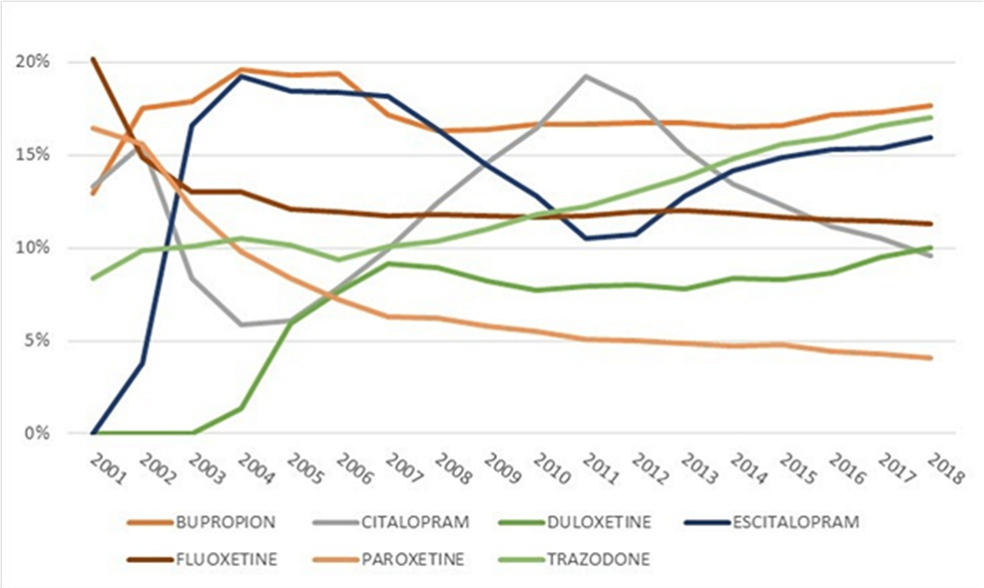
Antidepressants With More Than 5% Change in Use Over Study Period

There were 1,268,882 episodes (12.41%) of treatment with sertraline. Each of the top eight antidepressants was taken by more than 100,000 patients. The average duration of the antidepressant use was 215.97 days (standard deviation of 320.93, interquartile range (IQR) = 30 to 246). The average follow-up period (2.93 years) was longer than the average duration of antidepressant use as many patients had multiple antidepressants. Figure 3 shows the antidepressants with more than a 5% change in their market share per year. The use of citalopram peaked in 2011 and has fallen since then. The use of escitalopram peaked in 2004. Fluoxetine was the most common antidepressant initially but had a steady decline over the 17-year study period. These changes in antidepressant use have occurred during a period during which the published literature reported no difference in the effectiveness of antidepressants [12-15]. It is not clear why these changes occurred. 

Finding 2: high proportions of adverse outcomes in some subgroups

The proportion of adverse events associated with long-term (more than 100 days) antidepressant use is provided in Table 2. The study reported the proportions of adverse events among patients with major depression and taking antidepressants. Of particular interest was the proportion of suicide or self-harm among depressed teenagers on antidepressant treatment, which was 5.61%. This proportion is manyfold larger than for other age groups. The existence of wide variation in outcomes for subsets of patients highlights the need for further study of the comparative effectiveness of antidepressants.

**Table 2:** Adverse Events in Long-term Antidepressant Use

Adverse Events	Unique Long-term Episodes (N = 3,172,468)		Teens (Age 13-19) (N = 148,760)		Age 20-64 (N = 2,437,527)		Age 65+ (N = 586,181)	
	N	%	N	%	N	%	N	%
QT Interval	57,629	1.82	964	0.65	28,914	1.19	27,751	4.73
Fall & Fracture	176,395	5.56	5,208	3.50	89,571	3.67	81,616	13.92
Hyponatremia	46,493	1.47	165	0.11	20,329	0.83	25,999	4.44
GI Bleed	76,909	2.42	1,306	0.88	47,503	1.95	28,100	4.79
Elevated Liver Enzymes	63,115	1.99	758	0.51	45,931	1.88	16,426	2.80
Toxic Hepatitis	9,013	0.28	150	0.10	7,518	0.31	1,345	0.23
Blurred Vision	25,260	0.8	735	0.49	17,682	0.73	6,843	1.17
Constipation	148,661	4.69	3,938	2.65	86,811	3.56	57,912	9.88
Dry Mouth	6,677	0.21	40	0.03	3,754	0.15	2,883	0.49
Orthostatic Hypotension	21,625	0.68	730	0.49	8,594	0.35	12,301	2.10
Tachycardia	58,285	1.84	2,130	1.43	37,943	1.56	18,212	3.11
Urinary Retention	44,122	1.39	288	0.19	21,739	0.89	22,095	3.77
Weight Gain	77,220	2.43	2,386	1.60	67,480	2.77	7,354	1.25
Insomnia	366,224	11.54	7,134	4.80	274,566	11.26	84,524	14.42
Decreased Sexual Desire	14,908	0.47	49	0.03	14,129	0.58	730	0.12
Drug Interactions	22,973	0.72	680	0.46	16,106	0.66	6,187	1.06
Relapse to New Depression	516,539	16.28	28,496	19.16	395,747	16.24	92,296	15.75
Suicide or Self Harm	37,314	1.18	8,347	5.61	24,344	1.00	4,623	0.79

## Discussion

This study showed the procedure for construction of a cohort to evaluate the effectiveness of antidepressants using administrative claims data in the US. The construction of this cohort required several decisions about who is included (definition of depression), which medications are studied (most common medications and not new medications), the definition of symptom remission (based on diagnosis codes that include references to remission and patterns of abandoning antidepressants), and what can one expect from analysis of the cohort data (how long one can expect to follow patients, what differences in antidepressants can emerge). 

Definition of depression

A patient was included in the cohort if they ever took an antidepressant during the study period and also had a diagnosis of depression prior to the end of the study period. To identify if the patient had received an antidepressant, we used the Hedis (2019) NDC file [17]. Every year, the codes for antidepressants may change. To include a complete list, we used the 2019 list of codes to identify the generic names of antidepressants; and then reused these names to generate the earlier codes that were no longer valid. This method of defining the codes for antidepressants guaranteed that we would pick up the data coded in earlier years. Both patients with and without depression take antidepressants. Doctors prescribe antidepressants to treat anxiety, insomnia, chronic pain, panic disorders, fibromyalgia, migraine, obsessive-compulsive disorders, and a host of other "off-label" conditions, including migraine, menopause, attention-deficit/hyperactivity disorder, and digestive system disorders [20]. Two out of every three non-depression prescriptions for antidepressants were handed out under an off-label purpose. We excluded patients who receive antidepressants for non-depression diseases by requiring a prior diagnosis of depression. Despite our effort, some off-label uses of antidepressants may be included in the cohort. These situations can be further excluded in the analysis phase. In the analysis of the cohort, we required the dosage of antidepressants to eventually reach a therapeutic level. Off-label uses of antidepressants almost never reach the minimum therapeutic level set for depression. Therefore, we could rely on the dosage of the antidepressant to further reduce the inclusion of off-label antidepressant use in our data. 

An important decision in the design of the cohort centers around what diagnostic codes constitute the definition of diagnosis of depression. Over the years of our cohort, the International Classification of Diagnoses changed from version 9 to version 10. Both the codes for version 9 and version 10 were used to define depression. Table 3 shows the codes used by others to define the diagnosis of depression. The sensitivity of various case definitions is also reported in Table 3. Depression is mostly diagnosed in outpatient settings. Fiest et al. focused on inpatient data and, not surprisingly, the sensitivity of their definition is low [21]. In contrast, we define depression using both inpatient and outpatient codes. Fiest and colleagues describe various methods of defining depression, with the most restrictive definition being the most common. In contrast, we used the most inclusive and broadest definition of depression because the case definition is combined with the use of antidepressants, which research has shown to further improve its sensitivity [22].

**Table 3:** Sensitivity and Specificity of Different Definitions of Diagnosis of Depression Adapted from: Fiest et al., 2014 [21] ICD: International Classification of Diagnoses

Case definition	Codes	Sensitivity	Specificity
ICD 9 Restrictive	296.20 -.25, 296.30-.35, 300.4, & 311	28.93	99.66
ICD 9 Less Restrictive	Restrictive + 296.5, 296.6, 296.82 & 296.90	29.14	99.52
ICD 9 Most Inclusive	Less Restrictive + 309.0, 309.1 & 309.28	32.91	99.49
ICD 10 Restrictive	F32.0-32.9, F33.0-33.3, F33.8, F33.9, F34.1 & F41.2	34.17	99.55
ICD 10 Less Restrictive	Restrictive + F31.3-F31.6	34.59	99.52
ICD 10 Most Inclusive	Less Restrictive + F34.8, F34.9, F38.0, F38.1, F38.8, F39, F99	35.64	99.43

It is helpful to contrast the definition of this cohort with a claims-based analysis of monotherapy by Milea et al. [23]. Both Milea et al.'s and our cohort include patients of any age or gender based on the use of antidepressants. Milea et al. required no gap in utilization of health services exceeding 90 days, essentially excluding patients with low utilization of health services, such as young patients. In contrast, our approach included patients who had low healthcare utilization; as long as they were eligible for the health plan. Milea et al excluded patients with psychotic comorbidities or treatment 90 days prior to the start of antidepressants. We included these patients in the cohort but propose that future studies analyze this cohort separately on the impact of patients with psychiatric histories. Both Milea et al. and this cohort required data for a minimum of one year prior to and one year after the initial antidepressant. Milea et al. focused only on major depression. We also included adjustment disorders. Some clinicians treat adjustment disorders with antidepressants. Despite the inclusion of adjustment disorders in the cohort, future studies of this cohort should separately analyze those with adjustment disorders and those with major depression. 

A number of investigators have used minimum utilization criteria (e.g. two primary care visits or one hospitalization) to limit the cohort to patients who are regularly seen at the clinic or within an EHR. Minimum utilization within a clinic makes sense for dropping patients who are accidental users of the clinic, perhaps seeing other clinicians for their regular care. In the context of health plans, data from all clinics are sent into the same health plan, reducing the need to worry about the continuity of reports of care. In this context, eligibility for the health plan is far more important than minimal utilization criteria. Hence, our focus on eligibility at least one year prior and at least one year after the first purchase of the antidepressant. Furthermore, requiring minimal utilization could be problematic as well; patients (e.g. a teenager on antidepressants with no other illness) would be dropped from the data. Dropping these patients will distort study findings for an important subset of patients. Sometimes, the minimum utilization criterion is justified on the ground that classifying a patient as depressed based on a single diagnosis could be a rash decision, clinicians may have assigned the diagnosis as part of ruling out other diseases. Overwhelming evidence suggests that depression diagnoses are avoided and under-reported [24]. Patients, even those treated with antidepressants, may ask their doctor to list the diagnosis as insomnia, fatigue, anxiety, or other components of depression. It may be years after the start of antidepressants that the patient finally comes to terms with his/her illness. When depression is reported, it is an indication of the clinician and patient's deliberate decision. Therefore, depression is unlikely to be a rule-out diagnosis or to be entered without commitment to its treatment. In general, this cohort was organized with a broad definition of who is eligible so that the definition does not mask potential relationships in the data.

Definition of treatment variables

This cohort focused on the effectiveness of common antidepressants. Less common antidepressants include new antidepressants that are not widely used. The use of this cohort to evaluate the effectiveness of new antidepressants may not be reasonable. In this cohort, we can see the shift in the patterns of common antidepressant use in the period between 2001 and 2018. These data point to ongoing extensive experimentation in prescribing antidepressants. In retrospective data, the large variations in treatment utilization point to natural experiments embedded in the data. These events increase the usefulness of the cohort in detecting the comparative effectiveness of medications. The cohort also identified adverse events among patients with long-term antidepressant use. Of particular interest were the findings that the proportion of suicide or self-harm among depressed teenagers was manyfold larger than for other age groups. Future studies need to investigate whether the high rates of suicides and self-harm are caused by severe depression, inadequate treatment, or the use of wrong antidepressants. In recent years, a number of investigators have focused on the long-term effects of antidepressants [25]; and this should be of particular interest to regulatory agencies. Antidepressants were approved based on studies that examined short-term effects and long-term use remains controversial. 

Definition of the outcome variables

In this cohort, we can study both the short (within 100 days) and long-term outcomes of depression. In clinical studies of the effectiveness of antidepressants, the main outcome variable is patient-reported remission of depression symptoms. Unfortunately, this outcome is not available in claim-based data. Other investigators have used (a) switch in medication and (b) duration of use of antidepressants as a proxy for remission [26-31].

We designed a surrogate index to replace self-reported symptom remission. This index relied on four variables: duration of use, reaching therapeutic dose, switch/augmentation of antidepressant, and prior use of antidepressant. Alemi and colleagues provide the probability of remission at various combinations of these four measures, and, furthermore, they show that these four measures have a nearly perfect (area under the receiver operating curve of 0.93) for predicting symptom remission [19]. Therefore, when patient-reported remission information is missing, then the combined use of these four measures may be a reasonable surrogate measure for patient-reported symptom remission.

The key variable in the index was the definition of a switch, as other variables such as duration and reaching dosage are affected by an early switch. A switch is said to have occurred if the current antidepressant is stopped and another antidepressant in the same, or in a different, family was started within 60 days of stopping the original medication. A switch in treatment has also occurred when the patient stops an antidepressant and receives an electric shock treatment (CPT code 90870, Single Seizure; or 90871, Multiple Seizures, per day, ICD10 PCS Code GZB2ZZZ), a treatment of last resort for depressed patients [32]. A change to a generic brand is not considered a switch, nor are adjustments in the dosage of a medication considered a switch in medication.

Limitations

The cohort relies on observational data in administrative claims. The limitations for claims data include the accuracy of billing codes, the lack of follow-up and outcome data, limited granularity, and the risk of unmeasured confounding [33]. In observational data, one has to reduce confounding. We encourage the use of stratification to control for spurious correlations in observational data [34]. The use of antidepressant patterns as a surrogate for remission is novel and additional data are needed to further confirm that it is a reasonable surrogate for patient-reported symptom remission. The most recent data in this cohort is more than four years old. A great deal can change in prescription patterns in four years. During these years, for example, new generation antidepressants have been put into practice, although very few patients are receiving these antidepressants. A cohort study of millions of patients is a time-consuming activity (data needs to be submitted from practices to insurers, maintained in tables, curated for analytical studies, variables and measures defined, and cohort organized) and by the nature of the activities needed, the analysis will always lag several years. Furthermore, even if the analysis files are readily available, few data points are available on new antidepressants. One has to wait for new antidepressants to be prescribed in significant numbers to have sufficient power for analysis. The cohort described in this paper does not address new antidepressants, which are not common in current prescriptions. The focus is on the 15 most common antidepressants. As antidepressant prescription patterns change, more data will become available on the new medications. Future studies can include more recent data and address medications ignored in the current research plan. These future studies can benefit from the procedures described here; even though our data may be too old or too incomplete to be useful to future studies

## Conclusions

This study shows the procedures that can be used to organize a post-market release evaluation of the effectiveness of common antidepressant medications. It is not intended to report any particular findings from the analysis of the cohort. At the same time, it is useful to show the potential findings that could emerge from an analysis of the cohort. Those potential findings should be considered hypotheses/questions that could be answered in future analyses of the data on the cohort. This cohort can help regulatory agencies, the scientific community, clinicians, and patients examine which antidepressant is best and for whom. The details of the construction are included so that future investigators can design their own cohorts of patients using their access to claims data.

## Appendices

**Table 4:** Codes for depression

Code	Code Type	Description
293.83	ICD-9 CM	Mood disorder in conditions classified elsewhere
296.20	ICD-9 CM	Major depressive disorder, single episode, unspecified
296.21	ICD-9 CM	Major depressive disorder, single episode, mild
296.22	ICD-9 CM	Major depressive disorder, single episode, moderate
296.23	ICD-9 CM	Major depressive disorder, single episode, severe, without mention of psychotic behavior
296.24	ICD-9 CM	Major depressive disorder, single episode, severe, specified as with psychotic behavior
296.25	ICD-9 CM	Major depressive disorder, single episode, in partial or unspecified remission
296.26	ICD-9 CM	Major depressive disorder, single episode, in partial or unspecified remission
296.30	ICD-9 CM	Major depressive disorder, recurrent episode, unspecified
296.31	ICD-9 CM	Major depressive disorder, recurrent episode, mild
296.32	ICD-9 CM	Major depressive disorder, recurrent episode, moderate
296.33	ICD-9 CM	Major depressive disorder, recurrent episode, severe, without mention of psychotic behavior
296.34	ICD-9 CM	Major depressive disorder, recurrent episode, severe, specified as with psychotic behavior
296.35	ICD-9 CM	Major depressive disorder, recurrent episode, in partial or unspecified remission
296.36	ICD-9 CM	Major depressive disorder, recurrent episode, in full remission
296.82	ICD-9 CM	Atypical depressive disorder
298.0	ICD-9 CM	Depressive type psychosis
300.4	ICD-9 CM	Dysthymic disorder
309.0	ICD-9 CM	Adjustment disorder with depressed mood
309.1	ICD-9 CM	Prolonged depressive reaction
309.28	ICD-9 CM	Adjustment disorder with mixed anxiety and depressed mood
309.3	ICD-9 CM	Adjustment disorder with disturbance of conduct
309.89	ICD-9 CM	Other specified adjustment reactions, other
311	ICD-9 CM	Depressive disorder, not elsewhere classified
625.4	ICD-9 CM	Premenstrual tension syndromes
642.44	ICD-9 CM	Mild or unspecified pre-eclampsia, postpartum condition or complication
648.40	ICD-9 CM	Other mental disorders complicating pregnancy, unspecified trimester
648.41	ICD-9 CM	Maternal mental disorders, with delivery
648.42	ICD-9 CM	Maternal mental disorders, with delivery, with current postpartum complication
648.43	ICD-9 CM	Maternal mental disorders, antepartum
F06.30	ICD-10CM	Mood disorder due to known physiological condition, unspecified
F06.31	ICD-10CM	Mood disorder due to known physiological condition with depressive features
F06.32	ICD-10CM	Mood disorder due to known physiological condition with major depressive-like episode
F06.33	ICD-10CM	Mood disorder due to known physiological condition with manic features
F06.34	ICD-10CM	Mood disorder due to known physiological condition with mixed features
F31.71	ICD-10CM	Bipolar disorder, in partial remission, most recent episode hypomanic
F31.72	ICD-10CM	Bipolar disorder, in full remission, most recent episode hypomanic
F32.0	ICD-10CM	Major depressive disorder, single episode, mild
F32.1	ICD-10CM	Major depressive disorder, single episode, moderate
F32.20	ICD-10CM	Major depressive disorder, single episode, severe without psychotic features
F32.3	ICD-10CM	Major depressive disorder, single episode, severe with psychotic features
F32.4	ICD-10CM	Major depressive disorder, single episode, in partial remission
F32.5	ICD-10CM	Major depressive disorder, single episode, in full remission
F32.81	ICD-10CM	Premenstrual dysphoric disorder
F32.89	ICD-10CM	Atypical depressive disorder
F32.9	ICD-10CM	Major depressive disorder, single episode, unspecified
F33.0	ICD-10CM	Major depressive disorder, recurrent, mild
F33.1	ICD-10CM	Major depressive disorder, recurrent, moderate
F33.2	ICD-10CM	Major depressive disorder, recurrent severe without psychotic features
F33.3	ICD-10CM	Major depressive disorder, recurrent, severe with psychotic symptoms
F33.40	ICD-10CM	Major depressive disorder, recurrent, in remission, unspecified
F33.41	ICD-10CM	Major depressive affective disorder, recurrent episode, severe degree, without mention of psychotic behavior
F33.42	ICD-10CM	Major depressive disorder, recurrent, in full remission
F33.9	ICD-10CM	Major depressive disorder, recurrent, unspecified
F34.1	ICD-10CM	Dysthymic disorder
F43.21	ICD-10CM	Adjustment disorder with depressed mood
F43.23	ICD-10CM	Adjustment disorder with mixed anxiety and depressed mood
F43.24	ICD-10CM	Adjustment disorder with disturbance of conduct
F43.8	ICD-10CM	Other specified adjustment reactions
N94.3	ICD-10CM	Premenstrual tension syndrome
O14.05	ICD-10CM	Mild or unspecified pre-eclampsia, postpartum condition or complication
O14.95	ICD-10CM	Mild or unspecified pre-eclampsia, postpartum condition or complication
O15.2	ICD-10CM	Mild or unspecified pre-eclampsia, postpartum condition or complication
O90.6	ICD-10CM	Postpartum mood disturbance
O99.340	ICD-10CM	Other mental disorders complicating pregnancy, unspecified trimester
O99.341	ICD-10CM	Other mental disorders complicating pregnancy, first trimester
O99.342	ICD-10CM	Other mental disorders complicating pregnancy, second trimester
O99.343	ICD-10CM	Other mental disorders complicating pregnancy, third trimester
O99.344	ICD-10CM	Other mental disorders complicating childbirth
O99.345	ICD-10CM	Other mental disorders complicating the puerperium
